# Predictability of frailty index and its components on mortality in older adults in China

**DOI:** 10.1186/s12877-016-0317-z

**Published:** 2016-07-25

**Authors:** Fang Yang, Danan Gu

**Affiliations:** 1 Department of Social Work, School of Sociology and Political Science, Shanghai University, Shanghai, China; 2 United Nations Population Division, Two UN Plaza, DC2-1910, New York, NY 10017 USA

**Keywords:** Frailty index, Frailty components, Mortality, Older adults, CLHLS, China

## Abstract

**Background:**

Frailty represents an increased vulnerability to external stressors due to decreased physiological reserve and dysfunction in multiple bodily systems. The relationship between frailty and mortality has been well-documented in the literature. However, less is known about the predictive powers of frailty index and its components on mortality when they are simultaneously present. This study aimed to examine the predictive powers of frailty index and its multiple components on mortality in a nationally representative sample of older adults in China.

**Methods:**

We used a sample of 13,731 older adults from the 2008/2009 and 2011/2012 waves of the Chinese Longitudinal Healthy Longevity Survey (CLHLS). Frailty was measured using the cumulative deficit approach, and was constructed from 38 health variables (39 deficits). We selected 8 major sets of components: activities of daily living (ADL) (6 deficits), instrumental ADL (IADL) (8 deficits), functional limitations (5 deficits), overall cognitive functioning (1 deficit), chronic disease conditions (11 deficits), self-reported health (2 deficits), hearing and vision impairment (2 deficits), and psychological distress (1 deficit). Survival analysis was used to examine the roles of the frailty and its components in mortality.

**Results:**

Results showed that almost all the components of the frailty index (except chronic diseases) were significant predictors of mortality when examined individually. Among the components, ADL and IADL disabilities remained significant when considering all the components simultaneously. When the frailty and its components were simultaneously analyzed, the frailty remained a robust predictor of mortality across the age and sex groups, while most components lost their significance except ADL, IADL, and cognitive function components in some cases.

**Conclusions:**

Frailty measured by cumulative deficits has a stronger predictive power on mortality than its all individual components. ​ADL and IADL disability play a greater role in mortality than other components when considering all the components of frailty.

## Background

Frailty has received increasing academic attention in the field of gerontology and geriatrics in the past 30 years [1–4]. Frailty represents multifactorial vulnerability to external stressors due to decreased physiological reserve and dysregulation in multiple bodily systems [1, 3, 5–8]. Despite the heated discussion on this topic, there is no consensus regarding the measures of frailty [1, 3, 9]. However, there are two widely used models to measure frailty: one is the phenotype model and the other is the health deficit accumulation model. The former assesses frailty using five specific manifest indicators, including unintentional weight loss, exhaustion, low physical activity, slowness, and weakness [1]. The latter measures frailty using the proportion of present deficits to all the possible health deficits in physical, functional, and psychosocial domains for a given person, or frailty index [3, 7, 10]. Different from the phenotype model, frailty index focuses more on the aggregate decline in psychosocial and physical functioning, and it is a promising proxy measure for biological aging [5, 6, 9, 11].

Research has demonstrated that frailty is a significant predictor of a variety of outcomes, such as falls, hospitalization, health change, and mortality, and the relationships between frailty and the outcomes are independent of various confounders and chronological age [5–7, 10, 12–14]. Taking mortality for example, the frailty-mortality association has been well-established in the literature. For instance, empirical research has shown that frailty index predicts mortality in general older populations and even in centenarians, for both women and men [2, 6, 10, 12], and in older adults from different cultural backgrounds [6, 9, 12, 15].

Regarding the variables used to construct frailty index, several criteria have to be met. The variables should cover a range of health deficits, the prevalence of the overall deficits should generally increase with age, and they should not saturate too early [16]. So far, the following components have been commonly used to construct frailty index: cognitive impairment, chronic illness, disability in activities of daily living (ADL), disability in instrumental activities of daily living (IADL), functional limitation, self-rated health, chronic disease conditions, hearing and vision losses, and psychological distress [2, 3, 5–7, 12, 13, 16]. Some studies have also included biomarkers in the index [17, 18]. However, this cumulative deficit approach does not necessarily require the same number or the same set of variables for construction of the index because the indexes could yield comparable results as long as the variable list includes the main health domains [4, 16, 19].

One advantage of frailty index is that it incorporates a variety of health deficits whose individual contributions to health and mortality risks may be too small to be detectable, and a combination of these deficits into a single index could enhance its explanatory power [10, 20]. However, some studies have also showed that the significance of some components clustered in a few major domains of the frailty index in determining mortality is noticeable. For instance, Gu found that when excluding eight IADL disability items from the frailty index list that included 39 frailty items from a nationwide survey in China, the predictive power of the frailty index from 31 remaining deficits on  mortality was reduced, and that it was also the true for the case where a frailty index was generated from the randomly selected half of the variables from the list of thirty-nine items. In all other cases, the reduced predictive power was small [19]. Theou and colleagues found that the frailty index including disability and co-morbidity increased its predictive power for mortality than the index excluding disability and co-morbidity [20]. To the best of our knowledge, these studies are among the first that provided empirical evidence about comparisons of relative predictive power for mortality between the frailty index and its components, which have improved our understanding about the frailty index and its components in predicting subsequent mortality at old ages.

However, no studies have systematically examined the predictive power of the frailty index on mortality compared to that of its components in the literature. Researchers have argued that the frailty index is a “macroscopic variable” that represents general organism damage of an individual rather than any specific health deficiency [3, 5]. Thus, despite the well-established relationship between the frailty index and mortality, it remains largely unknown which component of the frailty index is significantly associated with mortality and whether the frailty index still is a significant predictor of mortality in presence of its components, and vice versa. The current study aims to address these research gaps. More specifically, we aim to examine the specific association between each component of the frailty index and mortality, and the association between the frailty index and mortality in a nationally representative sample of older adults in mainland China (hereafter China). Despite increasing research on frailty in developed countries, it is under-studied in developing countries, like China. Examination on frailty in Chinese older adults could better inform the eldercare policy-making and interventions.

## Methods

### Study sample

This study used the 2008/2009 and 2011/2012 waves of the Chinese Longitudinal Healthy Longevity Survey (CLHLS). The CLHLS was initiated in 1998, and is the first national longitudinal project to investigate the determinants of health and longevity of older adults in China from a multidisciplinary perspective. One significant characteristics of the project is that it has the largest sample of the oldest old so far from a developing country, which could enable a better understanding of human healthy longevity. The CLHLS was conducted through in-home interviews with extensive information collected in half of the randomly selected cities/counties in 22 out of 31 provinces in China. The CLHLS aims to interview all centenarians in the sampled cities/counties. Age of each centenarian was validated based on various sources whenever available, including birth certificate, genealogical documents, household booklet, and ages of their children and siblings [21]. For each centenarian interviewee, one nearby octogenarian and one nearby nonagenarian (living in either a community or an institution) with pre-designated age and sex were randomly interviewed; and for every three centenarians, four participants aged 65–79 (living in either a community or an institution) were randomly chosen based on a random code assigned to the centenarian in the same cities/counties where centenarians were living at the time of survey. Each respondent provided a written informed consent to indicate his/her willingness to participate in the CLHLS. The informed consent was signed by the next-of-kin if the respondent was not able to write. The CLHLS has collected information on demographics, family and household characteristics, health behaviors and lifestyles, economic resources, self-rated health and life satisfaction, cognitive functioning, physical examination, activities of daily living and instrumental activities of daily living, and chronic illness. More details about the sampling procedures and data collected can be found elsewhere [21–23], thus not presented herein.

In the 2008/2009 wave, the total number of participants was (*N* = 16,563). The response rate was 98 %. Of the total participants, 8,286 (50.03 %) were re-interviewed in the 2011/2012 wave, 5,445 (32.87 %) died before the 2011/2012 follow-up, and the rest of the participants (17.10 %) were lost to follow-up. A comparison between those lost to follow-up and those included in the analysis found that participants who were older, Han-ethnicity, living in the urban areas, financially independent, or frailer were more likely to be lost to follow-up in the 2011/2012 wave, and those who were married and currently smoking were less likely to be lost to follow-up (results available upon request). The dates of death for deceased participants were collected from various informants, such as next of kin, neighbors, neighborhood committees, and death certificate whenever available. Systematical assessments for the accuracy of age reporting, the randomness of attrition, the reliability, validity, and the consistency of numerous measures of health outcomes and mortality of the CLHLS were performed and the data quality was high [21–23].

### Measurements

#### Frailty index

In this study, frailty index was measured by the proportion of the number of health deficits presented to the total number of possible health deficits for a given person. The score range of the index was from 0 to 1 in this study, with higher scores denoting higher level of frailty. The indicator included 39 deficits from a total of 38 indicators encompassing self-rated health, cognitive functioning, ADL disability, IADL disability, functional limitations, hearing and vision impairments, chronic disease conditions, serious illness measured by being hospitalized or bedridden, psychological distress, and so forth. If an individual experiences more than one serious illness in the past 2 years, an additional deficit score is assigned to this person [2, 5, 6]. These indicators are similar to those used in previous research [2, 6, 10, 15]. Following the scoring method in the literature [2, 5, 6], each deficit was coded as 1 when it was present, and 0 when it was absent. However, as noted below, we did not use the 0 ~ 1 range for the index in regression models. Instead we used the number of deficits to measure the level of frailty of a given respondent for the purpose to be consistent with its components.

#### Components of the frailty index

We have selected following 8 major sets of the components: cognitive functioning, chronic diseases, ADL disability, IADL disability, functional limitations, self-rated health, hearing or vision impairment, and psychological distress, which have been frequently used in constructing frailty index in the literature [2, 3, 5–7, 12, 13, 16]. In the CLHLS, cognitive functioning was measured by a Chinese version of the Mini-Mental State Examination (MMSE) with a total score of 30, and respondents with a score of 23 or lower were considered as cognitively impaired [21–23]. Chronic diseases were measured by older adults’ self-reports from a list of 11 illnesses (e.g., diabetes, heart disease, stroke, hypertension, cancer). ADL disability and IADL disability were assessed by whether a respondent needed any assistance in performing six basic daily activities (e.g., bathing, dressing) and 8 activities that are important for independent living (e.g., cooking, shopping), respectively. Functional limitations were measured by five objective examinations, such as hand behind lower back, raising arms upright, standing from sitting a chair. Psychological distress was a single item used in constructing the frailty index. We defined a respondent to experience psychological distress if he or she often/always felt fearful/anxious, lonely/isolated, or useless. Self-rated health was constructed by two items including current self-assessed global health and the health status of the respondent compared with one year ago. Considering cerebrovascular and cardiovascular diseases are leading cause of mortality in China [24], we also generated another component by separating stroke and heart diseases from the rest of chronic diseases and explored their role in predicting mortality. Thus, in the analyses, there were nine sets of components in total.

#### Covariates

Based on previous literature [2, 5, 6, 10], we included the following covariates to obtain more robust results: age, ethnicity (Han vs. Non-Han), current urban/rural residence (urban vs. rural), marital status (currently married vs. no), education (one or more years of education vs. no schooling), lifetime primary occupation (white-collar occupation vs. all other occupations), economic independence (primary financial source from retirement wages or work vs. other sources), economic status (family economic status is rich or very rich in local community vs. others), co-residence with family members (yes vs. no), and health behaviors measured by current smoking (yes vs. no) and regular exercise (yes vs. no). Gender was not included as a covariate since all the analyses were stratified by sex.

#### Analytical strategy

First, we presented the sample descriptive statistics by age groups (ages 65–79, ages 80–89, ages 90–99, ages 100+) and sex. Second, we examined the predictive powers of the frailty index and each of its components on mortality by age groups and sex. Four regression models were designed. Model I controlled for age and ethnicity. Model II further controlled for all the other covariates listed in Table 1. Model III additionally controlled for all the other frailty components. Model IV added the frailty scores into Model II. To keep consistent with its components and better understand the association between the frailty and mortality in comparison to associations between its components and mortality, we used the number of cumulative deficits (ranging from 0 to 39), rather than the frailty index (ranging from 0 to 1) to represent the level of frailty of a given individual.

**Table 1:** Sample distributions by age and sex, CLHLS 2008/2009-2011/2012

		Total	Age groups					Sexes		
			65–79	80–89	90–99	100+	*p* ^*a*^	Women	Men	*p* ^*a*^
	Sample size	13,731	3,689	3,613	3,750	2,679		7, 872	5, 859	
	% of death, 2008/2009-2011/2012 (*n* = 5,445)	39.7	10.7	30.8	54.9	70.1	***	42.2	36.3	***
	Mean score of frailty (cumulative deficits)(0-39)	10.1	5.5	8.6	12.5	16.0	***	11.7	8.2	***
	Mean score of frailty index (0-1)	0.26	0.14	0.22	0.32	0.41	***	0.30	0.21	***
Deficit index components	% cognitive impaired	44.8	11.2	36.0	60.9	80.6	***	55.3	30.9	***
	Mean score of chronic diseases (0–11)	0.72	0.88	0.80	0.63	0.52	***	0.71	0.74	ns
	Mean score of ADL disability (0–6)	0.60	0.10	0.31	0.74	1.50	***	0.76	0.38	***
	Mean score of IADL disability (0–8)	3.57	0.85	2.84	4.88	6.47	***	4.26	2.64	***
	Mean score of functional limitations (0–5)	1.11	0.36	0.84	1.45	2.04	***	1.34	0.81	***
	Mean score of self-rated poor health (0–2)	1.44	1.37	1.45	1.47	1.47	***	1.46	1.40	***
	Mean score of hearing and vision impairments (0–2)	0.56	0.12	0.38	0.75	1.13	***	0.67	0.41	***
	% psychological distress	73.5	64.1	73.6	77.1	81.3	***	77.7	67.8	***
Covariates	% Men	42.7	53.1	50.7	41.7	18.9	***	NA	NA	NA
	% Han ethnicity	93.4	93.7	93.9	93.4	92.3	ns	93.2	93.8	ns
	% Urban residence	36.5	37.6	36.0	37.9	33.7	**	35.8	37.5	*
	% Married	30.9	65.5	34.0	13.4	3.2	***	17.5	48.8	***
	% 1 + year education	36.3	58.9	38.0	28.7	13.3	***	16.6	62.7	***
	% White collar occupation	6.4	11.0	6.3	5.3	2.1	***	2.2	12.1	***
	% Financial independence	23.6	48.5	21.0	14.3	5.9	***	12.7	38.3	***
	% Good economic status	13.2	12.6	13.3	14.5	12.2	*	12.1	14.8	***
	% Coresidence with family	83.3	85.7	79.3	80.3	89.4	***	82.6	84.1	*
	% Current smoking	17.9	26.6	20.0	14.6	7.4	***	5.7	34.2	***
	% Currently doing regular exercise	27.2	39.6	28.3	22.1	15.8	***	21.5	34.9	***

Note (1) unweighted. (2) all variables were measured at 2008/2009 except for % of death. (3) numbers in the parentheses of frailty and its components were the ranges or values of items/scores. (4) ^a^ *p* values of statistical tests were obtained from either Chi-square tests or ANOVA tests. (5) *NA* not applicable. (6) *n.s* not significant, *p < 0.05, **p < 0.01, ***p < 0.001

In the analyses, we used the Weibull hazard regression models to examine the predictive powers of frailty scores and its components on mortality. Mortality risk was the dependent variable of the survival analysis in the study, which was indicated by survival status and the duration of exposure to death. The number of days from the date of the 2008/2009 interview to the date of death or the date of the 2011/2012 follow-up interview was used to indicate the length of survival time. Those who were lost to follow-up in the 2011/2012 survey were dropped from the hazard analyses due to their unknown survival status and survival length, which leaves the total valid sample of 13,731. Previous research shows that excluding those lost to follow-up introduced little bias in the estimates [6], and our alternative approach including those lost to follow-up based on a multiple imputation yielded similar results (not shown). The multiple imputation assumed that those lost to follow-up had the same survival status and survival length from 2008/2009 to 2011/2012 as those who were not lost to follow-up if individuals in the former group had the same demographics, socioeconomic status, family/social support, health behaviors, and health conditions as those in the latter group.

We also checked the collinearity among components and the variance inflation factors (VIF) were all less than 2 when all components were presented in the model, which is below the criterion (VIF > =10) [25], indicating that the collinearity problem is not a big issue in the present study. The analyses were stratified by age and sex due to the well-established evidence on age and sex differences in frailty and mortality [2, 5, 6, 10, 15]. All the analyses were performed using Stata version 12.0.

## Results

**Fig. 1:**
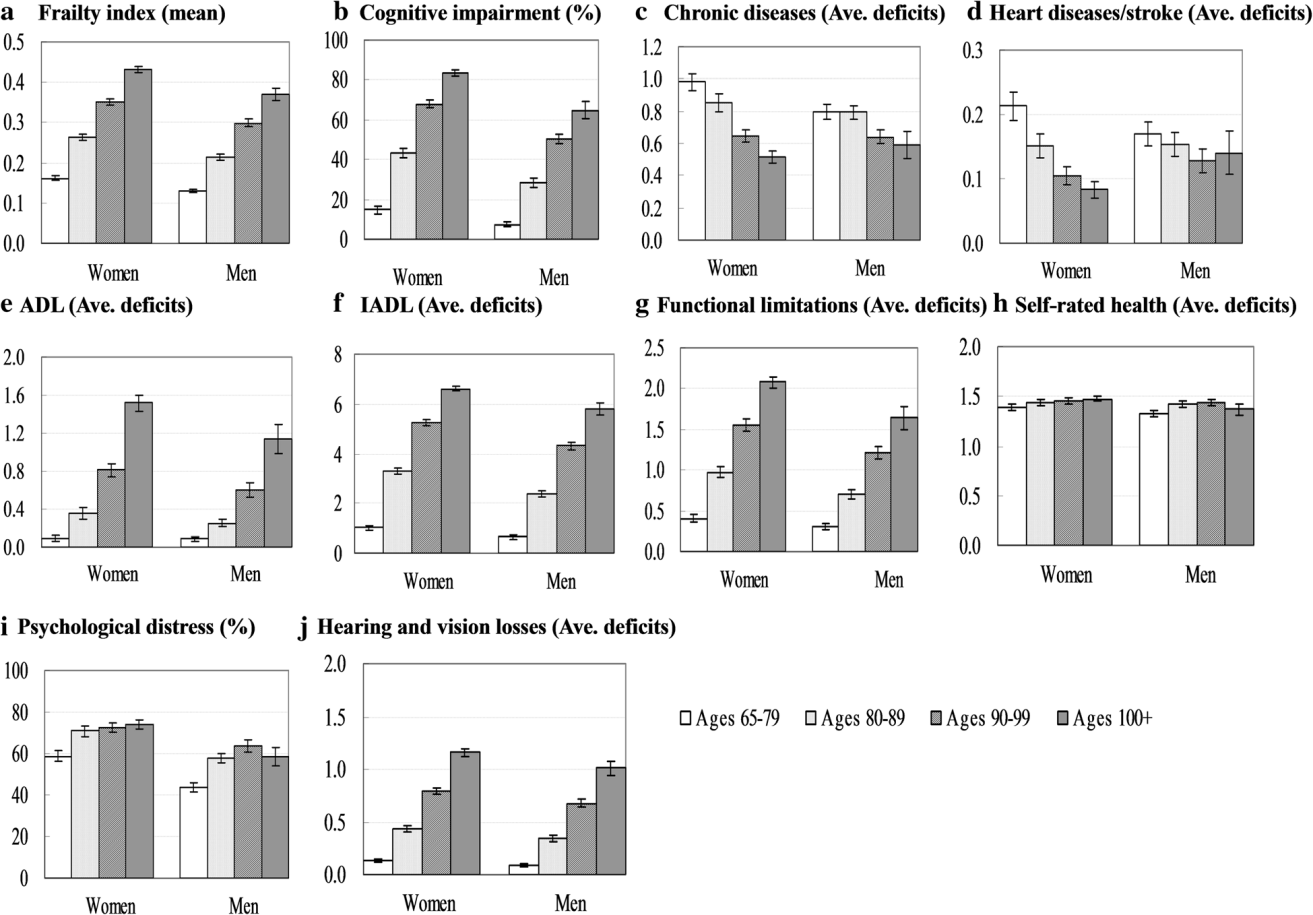
Mean of the frailty index and average deficits of its components by age and sex

Table 1 presents the sample distributions of the whole sample and by age and sex. The mean of the frailty index was 0.26 (∼ 10.1 deficits), and it increased with age from 0.14 (∼ 5.5 deficits) in ages 65–79 to 0.41 (∼ 16.0 deficits) in ages 100+. With respect to the frailty index components, almost all the components increased with age, except for chronic diseases. With respect to the covariates, the majority of respondents were Han-ethnicity (93.4 %) and coresided with their family members (83.3 %), 42.5 % of the respondents were male, 36.5 % lived in the urban areas, and 36.2 % attained 1+ year education. About 23.5 % of the respondents reported financial independence, 13.2 % in a good economic status, 17.7 % current smoking (17.7 %), and 27 % doing regular exercise. We also observed gender differences, such that men scored lower on the frailty index and almost all of its components than women, except for chronic diseases; that is, men were in better health than women. In addition, men reported better social economic status, and had a higher proportion in smoking and doing regular exercise than women. Figure 1 demonstrates the distributions of the frailty index and its components by age and sex.

Table 2 shows the hazard ratios of mortality from Weibull hazard models for the frailty and its components in men. Results (1^st^ column) show that a higher score of the frailty predicted a higher risk level of mortality across different age groups in Model I. For instance, each additional deficit of the frailty increased the odds of mortality by 5–10 %. In Model II, when adding other covariates, the mortality hazard ratios almost had no change. With respect to the frailty index components, results (columns 2 to 10) show that almost all the components significantly predicted a higher risk of mortality in all age groups with exceptions for chronic diseases and heart diseases or stroke in Model I. For example, each additional deficit in ADL disability, IADL disability, functional limitations, self-rated poor health, and hearing and vision impairment increased the odds of mortality by 14–47, 13–22, 14–34, 32–41, and 21–61 %, respectively. In Model II, these significant hazard ratios were only slightly reduced when controlling for other covariates. In Model III, ADL disability and IADL disability remained significant when adding all the other components of the frailty index, while other components turned to be non-significant. In Model IV, when the frailty was added into Model II (i.e., for each component model), the frailty remained significant across different age groups in all 9 sets of analyses for each of 9 components, whereas only the ADL disability and IADL disability components were significant for certain age groups. In a few cases (ages 80–89, 90–99), chronic diseases were negatively associated with mortality.

**Table 2:** Hazard ratios of 3-year mortality for frailty and its major selected frailty components by age group, CLHLS 2008/2009—2011/2012, Men

	Frailty scores (0–39)	Cognitive impairment (0 or 1)	Chronic diseases (0–11)	Heart diseases or stroke (0–2)	ADL disability (0–6)	IADL disability (0–8)	Functional limitations (0–5)	Self-rated poor health (0–2)	Hearing and vision impairments (0–2)	Psychological distress (0 or 1)
	(1)	(2)	(3)	(4)	(5)	(6)	(7)	(8)	(9)	(10)
*Model I set*										
Ages 65–79	1.10***	2.30***	1.14*	1.44**	1.47***	1.22***	1.34***	1.32**	1.61***	1.80***
Ages 80–89	1.08***	1.60***	1.06	1.18	1.32***	1.17***	1.25***	1.37***	1.35***	1.55***
Ages 90–99	1.08***	1.74***	1.03	1.11	1.30***	1.15***	1.23***	1.41***	1.36***	1.59***
Ages 100+	1.05***	1.23	1.08	1.13	1.14***	1.13***	1.14***	1.17	1.21**	1.28*
*Model II set*										
Ages 65–79	1.09***	2.05***	1.18**	1.49**	1.43***	1.19***	1.31***	1.29*	1.48**	1.69***
Ages 80–89	1.07***	1.43***	1.11*	1.33**	1.30***	1.16***	1.23***	1.34***	1.25***	1.41***
Ages 90–99	1.08***	1.58***	1.07	1.24*	1.29***	1.14***	1.22***	1.33***	1.28***	1.46***
Ages 100+	1.04***	1.18	1.08	1.10	1.12***	1.12***	1.12***	1.09	1.16*	1.23
*Model III set*										
Ages 65–79	NA ^a^	1.35	1.08	1.23	1.14	1.07	1.08	1.08	0.96	1.44*
Ages 80–89	NA ^a^	1.13	0.98	1.06	1.11*	1.13***	1.02	1.05	0.88	1.19
Ages 90–99	NA ^a^	1.24**	0.95	0.88	1.18***	1.06***	1.08**	1.08	0.98	1.15
Ages 100+	NA ^a^	0.89	1.01	1.00	1.07	1.11***	1.04	0.97	0.97	1.05
*Model IV set*										
Ages 65–79	---*** ^b^	1.16	0.96	1.00	1.04	0.99	0.97	0.95	0.90	1.32
Ages 80–89	---*** ^b^	1.00	0.89*	0.91	1.03	1.08**	0.96	0.99	0.88	1.09
Ages 90–99	---*** ^b^	1.11	0.87***	0.81*	1.11***	1.00	1.00	0.98	0.94	1.03
Ages 100+	---*** ^b^	0.84	0.96	0.89	1.01	1.08*^c^	0.99	0.89	0.93	1.00

Note. (1) numbers in the parentheses of frailty and its components were the ranges or values of items/scores. (2) Model I controlled for age and ethnicity. Model II further controlled for all other covariates listed in Table 1. Model III additionally controlled for all other frailty components. Model IV added frailty into Model II. (3) In the case of heart diseases or stroke, the variable of the number of chronic diseases excluded these two types of diseases. In the cases of frailty and all other frailty components, the variable “heart diseases or stroke” was excluded to avoid double control. (4) ^a^not applicable as frailty index was not included in Model III; ^b^frailty index was significant at *p* < 0.001 for all models in Model IV set; ^c^frailty was not significant in the model. (5) ^*^ *p* < 0.05, ^**^ *p* < 0.01, ^***^ *p* <0.001

Table 3 shows the hazard ratios of mortality from Weibull hazard models for the frailty and its components in women, with similar results as for men in Table 2. However, Table 3 shows that there were stronger links between ADL disability and cognitive impairment and mortality in women as compared to those in men (see Models III and IV).

**Table 3:** Hazard ratios of 3-year mortality for frailty and its major selected frailty components by age group, CLHLS 2008/2009—2011/2012, Women

	Frailty scores (0–39)	Cognitive impairment (0 or 1)	Chronic diseases (0–11)	Heart diseases or stroke (0–2)	ADL disability (0–6)	IADL disability (0–8)	Functional limitations (0–5)	Self-rated poor health (0–2)	Hearing and vision impairments (0–2)	Psychological distress (0 or 1)
	(1)	(2)	(3)	(4)	(5)	(6)	(7)	(8)	(9)	(10)
*Model I*										
Ages 65–79	1.10***	2.24***	1.15	1.15	1.51***	1.23***	1.25***	1.33*	1.35	2.05***
Ages 80–89	1.09***	1.77***	1.05	1.38**	1.36***	1.19***	1.27***	1.37***	1.48***	1.41**
Ages 90–99	1.07***	1.85***	0.99	1.04	1.23***	1.16***	1.17***	1.40***	1.40***	1.30***
Ages 100+	1.06***	1.55***	1.01	1.20*	1.16***	1.14***	1.14***	1.24***	1.31***	1.30***
*Model II*										
Ages 65–79	1.10***	2.09***	1.20*	1.28	1.47***	1.20***	1.24***	1.23	1.22	1.86**
Ages 80–89	1.09***	1.75***	1.06	1.47***	1.37***	1.19***	1.27***	1.32***	1.46***	1.34*
Ages 90–99	1.07***	1.77***	0.99	1.06	1.22***	1.15***	1.16***	1.34***	1.35***	1.20*
Ages 100+	1.06***	1.49***	1.01	1.19	1.16***	1.12***	1.13***	1.22***	1.28***	1.26***
*Model III*										
Ages 65–79	NA ^a^	1.73**	1.09	0.98	1.31**	1.11*	0.94	0.91	0.75	1.74*
Ages 80–89	NA ^a^	1.22	0.96	1.09	1.18***	1.10***	1.04	1.11	1.14	1.02
Ages 90–99	NA ^a^	1.44***	0.95	0.95	1.14***	1.07***	1.02	1.09	1.07	0.86
Ages 100+	NA ^a^	1.08	0.97	1.07	1.12***	1.04*	1.03	1.05	1.10*	1.04
*Model IV*										
Ages 65–79	---*** ^b^	1.42	0.97	0.83	1.16	1.01	0.87	0.85	0.76	1.38
Ages 80–89	---*** ^b^	1.22*	0.85***	0.92	1.10*	1.02	0.97	0.93	1.07	0.98
Ages 90–99	---*** ^b^	1.29***	0.86***	0.81*	1.07**	1.03	0.95*	1.02	1.05	0.86
Ages 100+	---*** ^b^	1.08	0.90**	0.96	1.07**	1.00	0.98	0.97	1.04	0.97

Note. (1) numbers in the parentheses of frailty and its components were the ranges or values of items/scores. (2) Model I controlled for age and ethnicity. Model II further controlled for all other covariates listed in Table 1. Model III additionally controlled for all other frailty components. Model IV added frailty into Model II. (3) In the case of heart diseases or stroke, the variable of the number of chronic diseases excluded these two types of diseases. In the cases of frailty and all other frailty components, the variable “heart diseases or stroke” was excluded to avoid double control. (4) ^a^not applicable as frailty index was not included in Model III; ^b^frailty index was significant at *p* < 0.001 for all models in Model IV set. (5) ^*^ *p* < 0.05, ^**^ *p* < 0.01, ^***^ *p* <0.001

## Discussion

Frailty is an important concept in aging research and practice, and it is a robust predictor of various health outcomes. However, less is known about the effects of the frailty index and its components on mortality among different older age groups, especially in developing countries. This study extended previous research and compared the predictive powers of the frailty index and its components on mortality in a nationally representative sample among different older age groups in China where mortality is relatively high and less resources are available to improve health for elderly population.

With respect to the predictive powers of frailty index components on mortality, we found that most components (except for chronic diseases) significantly predicted mortality when considered individually. These findings are consistent with previous studies [26–33]. The possible reasons for the non-significant role of chronic diseases might be the underreport of the chronic diseases, especially in rural areas of China, mainly due to underdeveloped local health service system or older adults’ poor health literacy [23]. Another possible reason is that we did not consider the severity of chronic diseases, which is also an important factor for mortality [34]. Considering cerebrovacular and cardiovascular diseases are leading causes of death in China [24], we examined the role of heart diseases or stroke in mortality, and the results were similar to those of chronic diseases covering a wider range of illnesses. Future research is warranted to shed light on this issue with more information on chronic diseases.

When considering all the components simultaneously, only ADL disability and IADL disability remained significant for both men and women, and cognitive impairment remained significant for women, whereas other components were not significant. These findings suggest that cognitive impairment (only for women), ADL and IADL disability (for both men and women) could partially explain the effects of other components on mortality. In other words, other components play a trivial role in determining mortality and will lose their predictivity when they are studied simultaneously with these three components. The greater predictive power of ADL and IADL disabilities on mortality compared to other index components is in line with previous findings [19, 20]. It is possible that ADL and IADL disabilities play a more important role in mortality, and may override the effects of other components, leading to non-significance of other components [28]. Previous research shows that other components (except self-rated health) will likely  cause ADL and IADL disabilities, which makes ADL and IADL disabilities be more proximal predictors to mortality [27, 35]. These findings suggest that some components may play a trivial role and lose its contribution to mortality when studied simultaneously. The significant role of cognitive impairment in predicting mortality in women indicates cognitive impairment is more lethal to women than to men in relation to mortality. Literature has shown that women tend to have a higher prevalence of cognitive impairment, disability, and chronic disease condition, depression, yet low mortality risk [36], which may cause a greater association between cognitive impairment and mortality in women than in men. Our finding is consistent with a recent study that found a greater attribution of cognitive impairment to mortality in women than in men [37].

In addition to the examinations on frailty index components, this study also shows that the frailty index remained a significant predictor of mortality across the age and sex groups when examining the frailty index only, which corroborates previous studies [2, 5–7, 10, 12–15]. We took a step further in the present study and compared the relative predictive powers of the frailty index and each of its components on mortality. We found that the frailty index remained a robust predictor of mortality, whereas the effects of its components were less consistent. These results provided strong empirical evidence for the argument that frailty is more powerful in predicting mortality than its individual components [19, 20]. This also highlights the importance of using the frailty index by accumulating different components into a single index when examining the relationship between physical health and mortality [10].

Surprisingly, chronic diseases were found to be a protective factor for mortality in both men and women when the frailty index and covariates were simultaneously controlled for. One possible reason might be due to the aforementioned underreport of the chronic diseases in older adults. Another speculation is that most people who reported to have chronic diseases got treated. Furthermore, older adults may change their unhealthy behaviors, or receive more social support and care from their family members when they were in illness, which might reduce the adverse impact of chronic diseases on mortality [38, 39]. One study using data from a small local area in China obtained a similar association between chronic disease conditions and mortality [37].

While stressing the advantages of the present research, its several limitations should be taken into account in interpreting the results. First, about 17 % of the participants interviewed in 2008/2009 were lost to follow-up in the 2011/2012 wave. Although the loss to follow-up is not at random, an alternative analysis that included those lost to follow-up did not alter the conclusion. Thus, our analysis would not bring a substantial bias in the conclusion. Second, similar to other surveys on older adults, there was a proportion of missing values due to increased age and declined cognitive abilities, proxies were used to reduce non-response, which might introduce biases when there was inconsistency between proxy ratings and those of the actual ratings of the participants. Further research is clearly warranted to shed light on this issue. Third, the present study assumed that the associations between each component and mortality and between the frailty index and mortality were linear. However, it is likely that the associations could be nonlinear, which deserves more research.

Our study also identifies some future research directions. For instance, this study only included variables in physical, functional, and psychosocial domains, but not biological or genetic domains. Recent research shows that biomarkers (e.g., CRP, IL-6, TNF-alpha) are potential physiological indicators of frailty [17, 18, 40]. It would be interesting to examine the relative predictive power of the biomarkers and frailty index on mortality. In addition, given that ADL and IADL disabilities seem to play a greater role in mortality compared to other components, the current construction of the frailty index approach that assumes equal weight for each deficit may deserve more research.

## Conclusions

Among the various components of the frailty index, ADL and IADL disabilities are stronger predictors of mortality than other components. Compared to its components, the collectively constructed frailty index is a universally consistent and robust predictor of mortality across the age and sex groups, whereas the effects of its components are less consistent and less robust. The findings provide empirical evidence that frailty index that accumulates mild-effect deficits of each component whose individual contribution to mortality might be too small, is a promising measure for global health of older adults.

## Abbreviations

ADL, activities of daily living; CLHLS, Chinese Longitudinal Healthy Longevity Survey; IADL, instrumental activities of daily living
